# Genome-Wide Association Mapping for Resistance to Leaf and Stripe Rust in Winter-Habit Hexaploid Wheat Landraces

**DOI:** 10.1371/journal.pone.0129580

**Published:** 2015-06-15

**Authors:** Albert Kertho, Sujan Mamidi, J. Michael Bonman, Phillip E. McClean, Maricelis Acevedo

**Affiliations:** 1 Department of Plant Pathology, North Dakota State University, Fargo, North Dakota, United States of America; 2 Department of Plant Sciences, North Dakota State University, Fargo, North Dakota, United States of America; 3 USDA-ARS, Small Grains and Potato Germplasm Research Unit, Aberdeen, Idaho, United States of America; USDA, UNITED STATES

## Abstract

Leaf rust, caused by *Puccinia triticina (Pt*), and stripe rust, caused by *P*. *striiformis* f. sp. *tritici (Pst*), are destructive foliar diseases of wheat worldwide. Breeding for disease resistance is the preferred strategy of managing both diseases. The continued emergence of new races of *Pt* and *Pst* requires a constant search for new sources of resistance. Here we report a genome-wide association analysis of 567 winter wheat (*Triticum aestivum*) landrace accessions using the Infinium iSelect 9K wheat SNP array to identify loci associated with seedling resistance to five races of *Pt* (MDCL, MFPS, THBL, TDBG, and TBDJ) and one race of *Pst* (PSTv-37) frequently found in the Northern Great Plains of the United States. Mixed linear models identified 65 and eight significant markers associated with leaf rust and stripe rust, respectively. Further, we identified 31 and three QTL associated with resistance to *Pt* and *Pst*, respectively. Eleven QTL, identified on chromosomes 3A, 4A, 5A, and 6D, are previously unknown for leaf rust resistance in *T*. *aestivum*.

## Introduction

Wheat leaf rust, caused by *Puccinia triticina (Pt)*, and wheat stripe rust, caused by *P*. *striiformis* f. sp. *tritici* (*Pst*), are important foliar diseases of wheat (*Triticum aestivum*) worldwide [1,2]. Genetic resistance is the preferred method of protecting against yield losses due to these diseases [3,4]. Resistance has been broadly categorized into all-stage resistance (also called seedling resistance) and adult-plant resistance (APR) [3]. Seedling resistance is expressed at all stages of plant growth, is mostly race-specific, and offers a high level of resistance; however it is easily overcome by changes in virulence of rust pathogens [2,5]. Conversely, APR is effective at later stages of plant growth and is mostly race-nonspecific and more durable [6]. The constant evolution of races of leaf rust and stripe rust pathogens with new virulences has rendered many wheat varieties susceptible [3,7–9]. Therefore there is a need to find new sources of resistance to manage these two important wheat diseases.

Currently more than 70 leaf rust resistance (*Lr*) and more than 50 stripe rust resistance (*Yr*) genes have been identified [10]. Most of these genes condition race-specific resistance in a gene-for-gene fashion and many have been overcome by the emergence of new races [11]. The most effective strategy of protecting wheat from rust is to deploy cultivars with both seedling and adult plant resistance genes. The use of seedling resistance is necessary to protect plants during early growth stages in production environments conducive to early-season disease development. Additionally, APR genes, such as high temperature adult plant resistance, is crucial for protecting plants at the critical stage of development and at high temperatures [3,12].

Previously, molecular markers linked to genes for resistance to leaf rust and stripe rust have been identified using bi-parental populations obtained by crossing resistant and susceptible wheat genotypes [13,14]. Though it has been successful, bi-parental QTL mapping generally requires years to develop a mapping population and gene discovery is limited to the genetic background of the two parents. Association mapping (AM) is an alternative to bi-parental linkage mapping that uses natural populations, thereby eliminating the need for developing mapping populations. AM is credited for detecting quantitative trait loci (QTL) with great resolution from populations of diverse origins [15]. AM uses linkage disequilibrium (LD) between alleles within diverse populations to identify markers associated with particular traits [16]. Recently, AM has been used to identify marker-trait associations in higher plants including disease resistance in potatoes [17] and wheat [18–23].

Wheat landraces are an important potential source of new resistance genes since relatively few landraces have been used in modern plant breeding [24]. The co-existence of rust pathogens and wheat may have resulted in the accumulation of diverse resistance in wheat [25]. Studies have demonstrated that wheat landraces can be a good source of resistance to leaf rust, stem rust, and stripe rust [21,26–29]. We therefore anticipate that new or underutilized genes for resistance to these rust pathogens may exist in winter wheat landraces. The objective of this research was to 1) identify potentially novel resistance QTLs to *Pt* and *Pst* in 575 winter wheat landrace accessions from the USDA National Small Grain Collection (NSGC) using an AM approach and 2) identify accessions with broad-spectrum resistance to races of the pathogens that are predominant in the U.S. northern Great Plains.

## Materials and Methods

### Wheat germplasm and pathogen races

A total of 567 winter wheat landrace accessions obtained through single plant selection from the *T*. *aestivum* core subset were provided by the NSGC located in Aberdeen, ID, U.S.A. The wheat accessions originated from 44 countries representing diverse geographic regions of the world. Five races of *Pt* (MCDL, MFPS, TDBG, THBL, and TBDJ), and one race of *Pst* (PSTv-37), representing prevalent races of the leaf rust and stripe rust pathogens in North Dakota were used to screen these accessions at the seedling stage in a greenhouse [30,31]. The virulence/avirulence profile of the rust races are based on reactions on seedlings of standard differentials used in the United States (Table 1)

**Table 1:** Virulence/avirulence profile of leaf rust and stripe rust pathogen races based on US differential set seedlings.

**Race**	**Virulent on genes**	**Avirulent on genes**
****PSTv-37**** ^a^	*6*,*7*,*8*,*9*,*17*,*27*,*43*,*44*,*Tr1*,*Exp2*	*1*,*5*,*10*,*15*,*24*,*32*,*SP*,*Tye*
****MCDL**** ^b^	*1*,*3*,*17*,*26*,*B*	*2a*,*2c*,*3ka*,*9*,*10*,*11*,*14a*,*16*,*18*,*24*,*30*
****MFPS**** ^b^	*1*,*3*,*3ka*,*10*,*14*,*17*,*24*,*26*,*30*,*B*	*2a*,*2c*,*9*,*11*,*16*,*18*
****THBL**** ^b^	*1*,*2a*,*2c*,*3*,*16*,*26*,*B*	*3ka*,*9*,*10*,*11*,*14a*,*17*,*18*,*24*,*30*
****TDBG**** ^b^	*1*,*2a*,*2c*,*3*,*10*,*24*	*3ka*,*9*,*11*,*14a*,*16*,*17*,*18*,*26*,*30*,*B*
****TBDJ**** ^b^	*1*,*2a*,*2c*,*3*,*10*,*17*,*14a*	*3ka*,*9*,*11*,*16*,*18*,*24*,*26*,*30*,*B*

^a^ *Pst* race nomenclature based on differentials lines in the United States (Wan & Chen, 2014)

^b^Four letter for *Pt* race nomenclature used in North America (Long & Kolmer, 1989).

### Phenotyping and data analysis

All the screening experiments were conducted at the North Dakota State University Agricultural Experiment Station Greenhouse Complex in Fargo, ND, U.S.A. The experiment was a randomized complete block design with three replicates and the entire experiment was repeated for each race of rust pathogen. Five seeds of each genotype were planted in 50-cell trays containing sunshine mix #1 (Sungro Horticulture Distribution Inc., Quincy, MI, USA) and slow-release commercial fertilizer (Osmocote 15-9-12, N-P-K, Everris NA Inc., Dublin, OH, USA) in a rust-free greenhouse set at 22°C /18°C (day/night) with 16-hour photoperiod. Susceptible checks ‘Little Club’ and ‘Avocet’ were included in each tray for leaf rust and stripe rust, respectively. Foliar fertilizer, Peat Lite 20-20-20, (Everris NA Inc., Dublin, OH, USA) was applied after seedling emergence and once per week thereafter. At 10 days after planting, seedlings at the two-leaf stage were spray inoculated with fresh rust urediniospores suspended in Soltrol-170 oil (Phillips Petroleum, Bartlesville, OK, U.S.A) at a rate of 0.01g/mL and then left to air dry.

Seedlings inoculated with *Pt* races were placed in a dark dew chamber for 16–24 hours at 20°C. The seedlings were then moved to a greenhouse until disease scoring. Infection types (ITs) were scored 12–14 days post-inoculation using the 0–4 scale [32] where IT 0 = no visible sign or symptom; 1 = small uredinia with necrosis; 2 = small to medium sized uredinia with green islands and surrounded by necrosis or chlorosis; 3 = medium sized uredinia with or without chlorosis; 4 = large uredinia without chlorosis. Accessions with ITs of 0 to 2 were considered resistant, whereas those with scores of 3 and 4 were considered susceptible.

Seedlings inoculated with PSTv-37 were placed in a clean dark growth chamber for 16–24 hours at 13°C and 98% humidity and then incubated in a growth chamber at 17°C/ 12°C (day/night) and 16-hour photoperiod. Disease reaction was assessed 16–18 days post-inoculation on a scale of 0-to-9 [7,12,32] where IT 0 = no visible signs or symptoms; 1 = necrotic or chlorotic flecks with no sporulation; 2 = necrotic and/or chlorotic blotches or stripes with no sporulation; 3 = necrotic and/or chlorotic blotches or stripes with only a trace of sporulation; 4, 5 and 6 = necrotic and/or chlorotic blotches or stripes with light, intermediate and moderate sporulation, respectively; and 7, 8 and 9 = abundant sporulation with necrotic and/or chlorotic stripes or blotches, chlorosis behind the sporulation area, and no chlorosis or necrosis, respectively. Plants with ITs 0–3 were considered resistant, 4–6 were considered intermediate and 7–9 were considered susceptible.

To account for multiple infection types in a single plant, the 0–4 Stakman disease rating scale [32] for leaf rust was converted to a linearized 0–9 disease scale [19] where rating 0–6 were considered resistant IT and 7–9 were considered as susceptible. Analysis of variance (ANOVA) was performed in SAS software 9.3 (SAS Institute, Cary, NC) before pooling IT data from two experiments. The median linear scale value for each accession, obtained from two experiments each with three replications, was used for association analysis.

### SNP marker genotyping and analysis

Five hundred and sixty seven winter wheat accessions were genotyped through the Triticeae Coordinated Agricultural Project using the Illumina iSelect 9K wheat array [33] at the USDA-ARS genotyping laboratory in Fargo, ND, U.S.A. A total of 5633 high quality polymorphic SNPs were selected and used for association analysis. Marker data are available at http://triticeaetoolbox.org/wheat/display_genotype.php?trial_code=NSGCwheat9K_winter_fac. Missing SNP data was imputed using fastPhase 1.3 [34] software with default settings. Markers with minor allele frequency (MAF) of less than 5% were removed, since the power of association with the phenotype are low for these alleles [35]. The genetic position of the SNP markers was estimated based on the wheat consensus map developed from Illumina iSelect 9K wheat array [33].

### Population structure and kinship

Population structure (Q-matrix) [36] was evaluated via principal component (PC) analysis using the PRINCOMP procedure in SAS 9.3 (SAS Institute, Cary, NC). PCs that explain 25% (PC_25_) and 50% (PC_50_) cumulative variation were used in mixed models for association analysis. An identity-by-state matrix (K-matrix) [37] estimated as a centered relatedness matrix in Gemma 0.92 [38] was used to estimate population relatedness.

### Association analysis

Four linear regression models were used to test for marker-trait associations. A Wilcoxon rank sum test was performed in SAS 9.3 using the npar1way procedure, for a Naïve model that did not account for population structure and kinship. Three other models that accounted for kinship (kinship, PC_25_ + kinship, PC_50_ + kinship) were analyzed in Gemma 0.92 [38]. The regression equation for mixed linear models used for association analysis takes the general form, *y* = Xß + Q*v* + I*u* + *e*, where *y* is a vector of recorded phenotype, X is a vector representing SNP genotype effects, ß is a vector of fixed effects due to the genotype, Q is a matrix estimating population structure, *v* is a vector of fixed effects arising from population effects, I is an identity matrix, *u* is a vector of random effects relating to co-ancestry and *e* is a vector of residual effects. The variances of random, *u* and residual, *e* effects are derived from the following assumptions; Var(*u*) = 2KV_g_ and Var(*e*) = V_R_, where K is a relative kinship matrix that compares the proportion of shared alleles between two individuals, V_g_ is the genetic variance and V_R_ is the residual variance [20,39]. The best model for each pathogen race was selected based on mean Squared Difference (MSD) between observed and expected *p*-values [40] since *p*-values of random markers follow a uniform distribution [39].

Marker-trait associations were considered significant at threshold of a positive false discovery rate (pFDR) of less than 0.1, a multiple comparison correction [41] calculated using the multtest procedure in SAS 9.3. Furthermore, stepwise regression was performed on all significant markers of each race using the REG procedure in SAS 9.3 in order to determine the minimum number of SNPs independently associated with disease resistance [21,42]. The selected markers from the stepwise regression explains the most phenotypic variation similar to variation explained by all markers considered together for each trait [42].

### *In silico* annotation of SNPs

Due to the incomplete genome sequence of *T*. *aestivum*, sequences of significant markers were searched for syntenic regions in related cereals whose genome sequence information are available. The putative biological functions of significant SNPs were determined by searching the sequences of the SNPs against protein sequences of sorghum (*Sorghum bicolor*), rice (*Oryza sativa*), and *Brachypodium distachyon* available in phytozome data base (http://www.phytozome.net). The homology search was performed using blastx.

## Results

### Seedling disease evaluations

**Fig 1:**
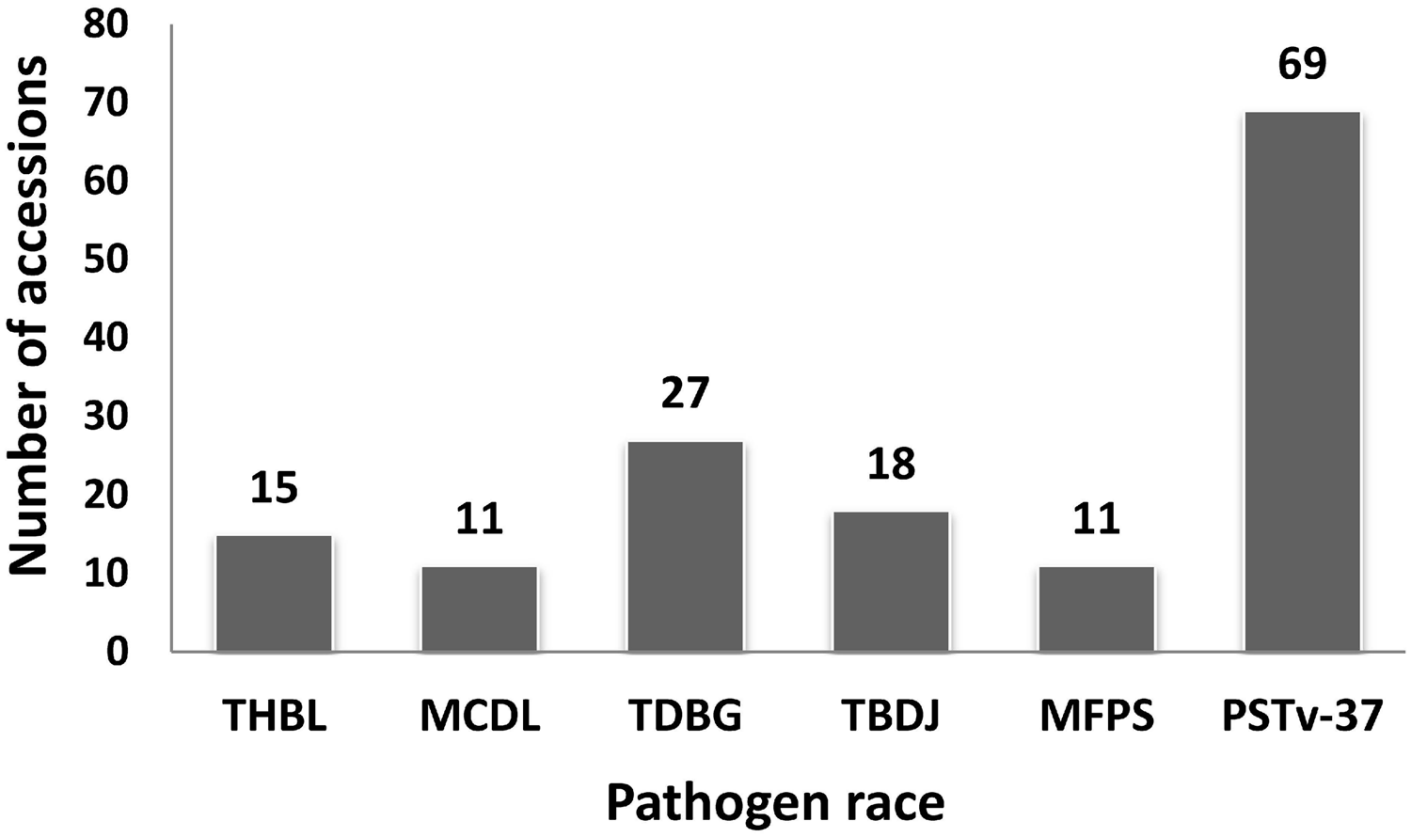
Number of accessions resistant to each race of *P*. *triticina* and *P*. *striiformis* f. sp. *tritici* tested. A total of 567 accessions were screened at the seedling stage with five races of *P*. *triticina* and one race of *P*. *striiformis* f. sp. *tritici*.

Phenotypic data was homogeneous based on the ANOVA of residuals of ITs for each pathogen race. Therefore, phenotypic data were pooled for each race and overall medians were used for association analysis (S1 Table). Most accessions were susceptible to the *Pt* races tested, with only 2.6%, 1.9%, 4.8%, 3.2%, and 1.9% accessions resistant to *Pt* races THBL, MCDL, TDBG, TBDJ and MFPS, respectively (Fig 1). For each of these races, the largest number of resistant accessions had median IT scores of 2. Disease reaction for *Pst* race PSTv-37 ranged from immunity (IT = 0) to complete susceptibility (IT = 9). Sixty-nine (12%), 73 (12.7%), and 433 (75.3) accessions were highly resistant (IT = 0–3) (Fig 1), moderately resistant (IT = 4–6) and susceptible (IT = 7–9), respectively. Among the accessions that were highly resistant, three accessions originating from Georgia, Egypt, and Chile showed immune infection type during both *Pst* experiments but were susceptible to the five races of *Pt*. Moreover, six accessions originating from Iran were highly resistant to all five races of *Pt* and the one race of *Pst* tested in this experiment (Table 2).

**Table 2:** Infection type of six accessions from Iran that show resistance to all five races of *Puccinia triticina* (*Pt*) and a race of *Puccinia striiformis* f. sp. *tritici* (*Pst*).

	*Pt* race	*Pt* race	*Pt* race	*Pt* race	*Pt* race	*Pst* race
Accession	MCDL	MFPS	THBL	TDBG	TBDJ	PSTv-37
**PI 621539**	2 (5)	2 (5)	2- (4)	;2 (2)	2/3 (6)	4
**PI 621674**	1 (2)	2- (4)	2+ (6)	2- (4)	1/2 (3)	6
**PI 622111**	2 (5)	1 (2)	2- (4)	1 (2)	12- (3)	1
**PI 622129**	1 (2)	1 (2)	1 (2)	1 (2)	1 (2)	1
**PI 622243**	1 (2)	2 (5)	2- (4)	2 (5)	12- (3)	1
**PI 622246**	1 (2)	1 (2)	1 (2)	1 (2)	1 (2)	1

(-) indicates slightly smaller uredinia than the standard, (+) indicates slighter larger uredinia, two infection types (IT) (such as 12-) indicates a mixed reaction on the same leaf, two IT separated by slash (such as 2/3) indicates varying reaction among seedling plants of the same accession (some seedlings are 2, other seedlings are 3). The linearized disease rating for leaf rust shown in parentheses was used in association analysis.

### Imputation, population structure, and model selection

**Fig 2:**
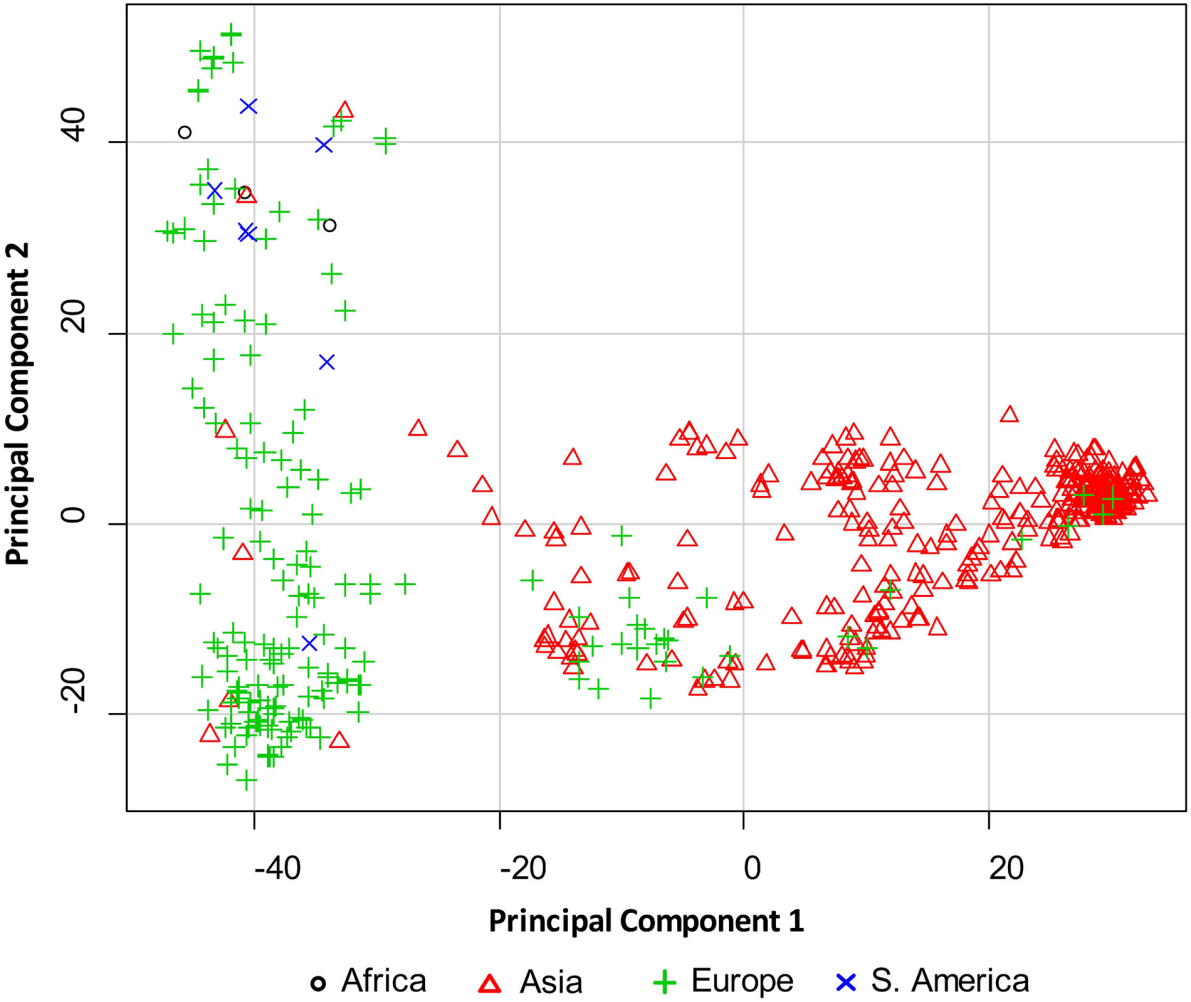
A graph showing two principal components obtained from 4234 polymorphic SNPs. PC1 and PC2 explain 19.41% and 5.22% variation, respectively.

A total of 5633 high quality SNPs were obtained from the 9K iSelect wheat SNP array (S2 Table). The 1.4% missing SNP data were imputed and 4234 SNPs with MAF of greater than 5% were selected for further analysis. Of the 4234 SNP markers, 3992 (94.3%) were previously mapped to the A (45.4%), B (43.7%), and D (5.2%) genomes of wheat [31]. Principal component (PC) analysis show that two and 20 PCs explain a cumulative 24.63% and 50.43% of the genotype variation, respectively. The first two PCs grouped the entries into two major clusters based on geographic location. One cluster contained accessions mainly from Asia and the other cluster had accessions mainly from Europe. The few accessions from Africa and South America grouped with accessions from Europe (Fig 2). Based on MSD values of the four linear regression models tested, no single model was best for all traits. The best models were as follows; Kinship for TDBG and THBL, PC_25_+Kinship for MCDL and MFPS and PC_50_+Kinship for TBDJ and PSTv-37 (Table 3).

**Table 3:** Mean square difference (MSD) for each disease race and model.

**Model**	**MCDL**	**MFPS**	**TBDJ**	**TDBG**	**THBL**	**PSTv37**
Naïve	6.23E-02	5.45E-02	9.39E-02	1.00E-01	8.11E-02	1.57E-01
Kinship	1.03E-04	2.80E-04	2.99E-04	**1.54E-04**	**1.48E-04**	5.32E-04
PC2+Kinship	**9.82E-05**	**2.69E-04**	2.69E-04	1.75E-04	1.63E-04	5.03E-04
PC20+Kinship	1.24E-04	2.84E-04	**8.78E-05**	2.24E-04	1.51E-04	**3.82E-04**

The best model was used to investigate SNP-rust resistance associations.

Numbers in bold indicate lowest mean square difference (MSD) and best model for each rust race.

### Marker-trait associations and *in silico* annotation of SNPs

**Fig 3:**
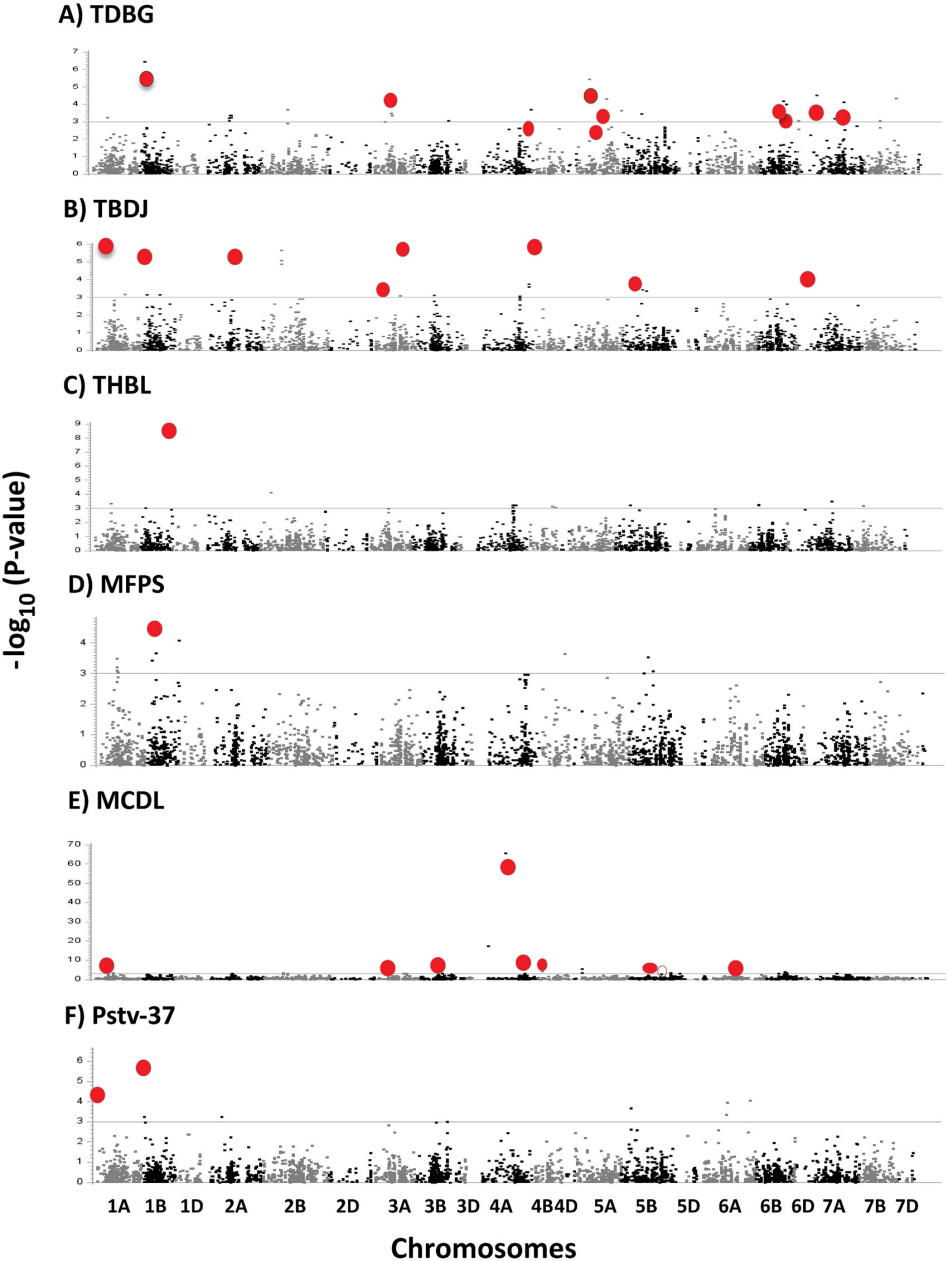
Manhattan plots showing *P* values across 21 wheat chromosomes for SNP markers associated with resistance to races of *P*. *triticina* (A-E) and *P*. *striiformis* f. sp. *tritici* (F). The horizontal black line indicates significant threshold at *p*-value = 0.001. SNPs included in stepwise regression are shown in red.

Statistically significant disease resistance QTL were determined by applying a FDR-adjusted *P* < 0.1 threshold (S3 Table). Thirty-four markers were significantly associated with resistance to race MCDL and were located on chromosomes 1A, 2B, 3A, 3B, 4A, 4B, 4D, 5B, 6A, and 6B (Table 4, Fig 3). Twenty-one of the 34 significant markers corresponded to a gene model based on a homology search against the protein sequences (Table 4). Eleven out of 34 significant markers fit into a regression model and together accounted for 38.2% of the phenotypic variation (Table 5). These markers are located at 11 QTL regions on chromosomes 1A, 3A, 3B, 4A, 4B, 5B, and 6A.

**Table 4:** Significant markers associated with resistance to each rust pathogen race.

**Trait**	**Marker**	**Chrom** ^a^	**cM** ^b^	**-log**_**10**_ **(p-value)**	**pFDR** ^c^	**SNP**	**MAF** ^d^	**Included in Stepwise Regression**	**Gene Annotation**
****MCDL****									
	IWA5702	1A	57.95	5.11	4.61E-03	[T/C]	9.17		Heat shock 70kDa protein
	IWA2768	1AS	72.53	3.19	8.60E-02	[T/C]	6.35	x	Protein of unknown function, DUF288
	IWA2887	2B	76.02	3.25	8.38E-02	[T/C]	5.11		Ankyrin repeat domain
	IWA295	2B	76.02	3.25	8.38E-02	[A/C]	5.11		
	IWA762	2B	76.02	3.25	8.38E-02	[A/G]	5.11		
	IWA5977	3AL	47.75	3.47	6.14E-02	[T/C]	39.15	x	
	IWA6244	3BL	71.14	5.8	1.65E-03	[T/C]	40.74	x	Arginyl-tRNA synthetase
	IWA4030	4A	4.06	17.18	9.20E-15	[A/G]	38.45		
	IWA2816	4A	74.81	65.56	3.79E-63	[A/G]	15.87		
	IWA3756	4AL	93.49	58.36	6.11E-56	[T/C]	15.7	x	Conserved oligomeric golgi complex component, COG2
	IWA7859	4AL	151.32	5.7	1.65E-03	[A/G]	31.22	x	Cation transporter/ATPase
	IWA2126	4B	16.37	4.22	2.78E-02	[T/C]	12.7	x	Peptidase family M1
	IWA3815	4D	52.44	5.23	4.05E-03	[T/C]	9.52		
	IWA286	4D	52.81	3.23	8.38E-02	[T/C]	9.17		
	IWA8375	5B	82.62	3.67	5.92E-02	[T/C]	39.51	x	
	IWA6694	5BL	168.74	3.22	8.38E-02	[A/G]	34.22	x	DNA binding
	IWA6737	6A	89.87	3.31	8.38E-02	[A/G]	40.74	x	DNA photolyase
	IWA185	6B	73.70	3.5	5.92E-02	[A/C]	11.82		leucine-rich repeat receptor-like protein kinase
	IWA3131	6B	73.70	3.5	5.92E-02	[T/C]	11.82		Coatomer WD associated region
	IWA3133	6B	73.70	3.5	5.92E-02	[A/G]	11.82		Coatomer WD associated region
	IWA5785	6B	73.70	3.8	5.92E-02	[A/C]	11.99		ATPase activity
	IWA6142	6B	73.70	3.5	5.92E-02	[A/G]	11.82		PRP38 family
	IWA6825	6B	73.70	3.5	5.92E-02	[A/G]	11.82		Coatomer WD associated region
	IWA6826	6B	73.70	3.5	5.92E-02	[T/C]	11.82		Coatomer WD associated region
	IWA7873	6B	73.70	3.5	5.92E-02	[A/C]	11.82		RNA recognition motif
	IWA8192	6B	73.70	3.5	5.92E-02	[T/C]	11.82		
	IWA596	6B	83.04	3.15	9.23E-02	[T/C]	41.8		
	IWA2121	Unk^e^	Unk	3.23	8.38E-02	[A/G]	9.17		Ribulose-phosphate 3 epimerase
	IWA2122	Unk	Unk	3.57	5.92E-02	[A/G]	10.05		Ribulose-phosphate 3 epimerase
	IWA287	Unk	Unk	3.57	5.92E-02	[A/G]	10.05		
	IWA397	Unk	Unk	3.09	9.92E-02	[A/C]	5.64	x	
	IWA55	Unk	Unk	3.57	5.92E-02	[A/G]	10.05		
	IWA6340	Unk	Unk	3.12	9.48E-02	[A/G]	6.88		Leucine rich repeat
	IWA8186	Unk	Unk	4.32	2.51E-02	[T/C]	40.04	x	Peptidase M16 inactive domain
****MFPS****									
	IWA7466	1BS	47.53	4.34	9.78E-02	[T/C]	5.82		
	IWA5418	1BS	47.53	4.34	9.78E-02	[T/C]	5.82	x	
****TBDJ****									
	IWA3160	1AS	51.12	6	1.54E-03	[T/C]	16.23	x	
	IWA435	1BL	30.47	5.17	3.53E-03	[T/C]	8.47	x	Protein kinase domain
	IWA574	2AS	103.39	5.18	3.53E-03	[T/G]	45.68	x	Protein phosphatase 2C
	IWA2887	2B	76.02	5.65	1.54E-03	[T/C]	5.11		Ankyrin repeat domain
	IWA295	2B	76.02	5.65	1.54E-03	[A/C]	5.11		
	IWA762	2B	76.02	5.65	1.54E-03	[A/G]	5.11		
	IWA2557	2B	76.37	4.86	5.77E-03	[A/G]	5.29		RhoGAP domain
	IWA3824	2B	77.53	5.07	3.91E-03	[A/G]	5.47		Phosphatidylethanolamine-binding protein
	IWA5977	3AL	47.75	3.43	9.16E-02	[T/C]	39.15	x	
	IWA3546	3AS	118.07	5.74	1.54E-03	[T/C]	7.76	x	IBR (In between ring finger) domain
	IWA285	4A	192.37	3.75	5.28E-02	[T/G]	11.29		
	IWA54	4A	192.37	3.75	5.28E-02	[T/G]	11.29		
	IWA8389	4A	192.37	3.59	7.06E-02	[A/G]	11.46		
	IWA2126	4B	16.37	5.7	1.54E-03	[T/C]	12.7	x	Peptidase family M1
	IWA8375	5B	82.62	3.43	9.16E-02	[T/C]	39.51	x	
	IWA619	6D2S	45.42	3.84	5.05E-02	[T/C]	5.11	x	
	IWA6340	Unk	Unk	4.75	6.68E-03	[A/G]	6.88		Leucine rich repeat
****TDBG****									
	IWA6290	1BL	30.47	6.43	1.37E-03	[A/G]	7.58	x	Glycerophosphoryl diester phosphodiesterase
	IWA7429	2A	91.42	3.36	8.92E-02	[A/G]	18.69		Protein phosphatase 2C
	IWA2195	2A	97.14	3.36	8.92E-02	[A/G]	18.69		OTU (ovarian tumor)-like cysteine protease
	IWA3924	2B	110.85	3.68	7.68E-02	[A/G]	9.88		Biological process
	IWA5006	3A	68.77	3.46	8.92E-02	[T/C]	6		
	IWA5005	3A	69.47	3.46	8.92E-02	[T/C]	6		ABC transporter
	IWA5786	3AS	72.50	3.37	8.92E-02	[A/G]	7.05	x	ABC transporter
	IWA1900	4AL	198.84	3.69	7.68E-02	[A/C]	5.82	x	F-box domain
	IWA7014	5A	53.71	5.43	6.82E-03	[A/G]	23.46	x	
	IWA3996	5A	87.89	3.36	8.92E-02	[T/C]	23.28	x	UDP-glucose pyrophosphorylase
	IWA2445	5A	122.72	4.3	3.70E-02	[A/C]	6.7	x	F-box domain
	IWA7361	5A	184.89	3.63	7.75E-02	[A/G]	8.29		Domain of unknown function DUF221
	IWA8395	5B	71.11	3.44	8.92E-02	[A/G]	5.47		Zinc finger, ZZ type
	IWA3699	6BS	95.67	4.17	4.10E-02	[A/G]	24.87	x	Zinc finger
	IWA7506	6BS	106.47	3.99	4.69E-02	[T/C]	8.29	x	myosin
	IWA7616	6D2S	69.20	4.52	3.70E-02	[A/G]	11.99	x	Ribonuclease II domain
	IWA5526	7AS	102.85	4.11	4.11E-02	[A/G]	8.47	x	GTP cyclohydrolase II
	IWA5000	7B	129.51	4.33	3.70E-02	[A/C]	6.17		TRAF-type zinc finger
****THBL****									
	IWA6512	1BS	140.72	8.51	1.29E-05	[T/C]	14.29	x	RecF/RecN/SMC N terminal domain
****PSTv-37****									
	IWA4240	1AL	0.00	4.27	5.98E-02	[A/G]	28.75	x	NB-ARC domain
	IWA7331	1BL	10.98	5.64	4.89E-03	[T/G]	20.28	x	WD domain, G-beta repeat
	IWA3526	6A	98.55	3.95	5.98E-02	[T/C]	8.11		
	IWA3527	6A	98.55	3.95	5.98E-02	[T/C]	8.11		
	IWA2416	6A	98.98	3.95	5.98E-02	[T/G]	8.11		Methyltransferase domain
	IWA8110	6A	99.63	3.95	5.98E-02	[T/G]	8.11		
	IWA6853	6A	193.68	4.02	5.98E-02	[A/G]	5.82		C-5 cytosine-specific DNA methylase
	IWA62	Unk	Unk	7.01	4.17E-04	[A/G]	13.23	x	

Markers labelled with ‘x’ were maintained after stepwise regression.

^a^Chrom = Chromosome;

^b^cM = Marker position on consensus map;

^c^pFDR = Positive false discovery rate;

^d^MAF = Minor allele frequency;

^e^Unk = Chromosomal location is unknown.

**Table 5:** Stepwise regression for each rust race.

**Rust race**	**No. of significant markers**	**Markers included in stepwise regression**	**% phenotypic variation explained**
MCDL	34	11	38.16
MFPS	2	1	0.015
TBDJ	17	8	32.62
TDBG	18	10	43.50
THBL	1	1	0.002
PSTv-37	8	3	29.43

Two SNP markers located on chromosome 1B were significantly associated with resistance to race MFPS (Table 4, Fig 3). One of the two markers fit into a stepwise regression. Seventeen SNP markers were associated with resistance to race TBDJ and were identified across the following chromosomes; 1A, 1B, 2A, 2B, 3A, 4A, 4B, 5B, and 6D (Table 4, Fig 3). Eight of the markers corresponded to protein sequences searched in other cereals (Table 4). Furthermore, eight of the 17 markers fit into a stepwise regression and accounted for 32.6% of the phenotypic variation (Table 5). The eight SNP markers are found at eight QTL regions and were identified on seven chromosomes 1A, 1B, 2A, 3A, 4B, 5B, and 6D.

Eighteen SNP markers detected on eleven chromosomes (1B, 2A, 2B, 3A, 4A, 5A, 5B, 6B, 6D, 7A, and 7B) were significantly associated with resistance to race TDBG (Table 4, Fig 3). Sixteen of the markers matched with protein sequences searched in three related cereals (Table 4). Ten out of the 18 significant markers fit into a stepwise regression and accounted for 43.5% of the phenotypic variation (Table 5). These 10 markers are spread among 10 QTL regions on chromosomes 1B, 3A, 4A, 5A, 6B, 6D, and 7A. One SNP marker identified on chromosome 1B was associated with resistance to race THBL (Table 4, Fig 3).

A total of seven SNP markers located on chromosomes 2B, 3A, 4B, and 5B were associated with resistance to both races MCDL and TBDJ (Table 4). Three out of the seven significant markers corresponded to the protein sequences searched in three cereals related to *T*. *aestivum* (Table 4). Three (IWA5977, IWA2126 and IWA8375) of the seven markers fit into a stepwise regression model.

A total of eight markers associated with resistance to PSTv-37 were located on chromosomes 1A, 1B, and 6A (Table 4, Fig 3). Four of the eight markers corresponded to protein sequences searched in other cereals (Table 4). Three out of eight significant markers fit into a stepwise regression model and accounted for 29.43% of the phenotypic variation (Table 5). These three markers were detected at three QTL regions on chromosomes 1A and 1B.

## Discussion

The evolution of new races of the leaf rust and stripe rust pathogens is a continuous threat to winter wheat production in the northern Great Plains of the United States. The available host genetic resistances is mostly race specific and easily overcome by pathogen evolution [3,10]. Therefore, there is need to find new sources of resistance and incorporate into adapted local cultivars. One major goal of this research was to identify winter wheat accessions possessing a wide spectrum of seedling resistance to leaf rust and to a predominant race of the stripe rust pathogen. Six landrace accessions (PI 621539, PI 621674, PI 622111, PI 622129, PI 622243, and PI 622246) that were resistant to all five races of *Pt* and one race of *Pst* tested at the seedling stage were identified. Geographic information available from NSGC established that all six accessions originated from Iran and were collected in the same year (NSGC 2010; http://www.ars.usda.gov/main/docs.htm?docid=2884). Four accessions were collected from Mazandaran province in northern Iran while the other two accessions were each collected from Tehran and Hamadan provinces located in northern and western Iran, respectively. Two of the accessions from Mazandaran (PI 622243 and PI 622246) were collected from the same exact location, but they exhibit differential reactions to races of *Pt* tested in this study. Moreover, the SNP genotype for both accessions was only 91% similar which suggests that these two accessions are not duplicates. Field evaluations at two locations in Washington, USA, where stripe rust is a major constraint to wheat production, showed these accessions to be highly resistant to the local stripe rust pathogen population (X. M. Chen, http://www.ars-grin.gov/npgs/acc/acc_queries.html). The identification of highly resistant accessions from Iran is not surprising as Iran is located in the Fertile Crescent, which is known as the center of origin and diversity of wheat [43]. Additionally, rust epidemics are common in Iran which could provide an opportunity for natural selection and maintenance of resistant genotypes by farmers [44,45]. This result also suggests that we might expect to obtain many accessions from Iran with resistance to leaf rust and stripe rust since the co-existence of rust pathogens and wheat is believed to result in accumulation of diverse resistance in wheat [25]. Though phenotypic and genotypic data show these accessions as different, allelism tests will be needed to determine if these accessions carry the same or different resistance genes.

Association mapping can produce spurious marker-trait associations if not corrected for population structure and relatedness among individuals [39,46]. Population structure analysis grouped the winter wheat accessions in this study into two major subpopulations. Therefore, we tested multiple models taking into consideration relatedness (K) and population structure (Q). Model analysis revealed that the best models are those that accounted for familial relatedness (K) and/or population structure (Q). Also, multiple testing corrections were used to further eliminate false positive associations. Initially, Manhattan plots of *p*-values showed many significant markers associated with resistance to each race of rust pathogen tested at a significant cutoff of *p*-value = 0.001. After multiple testing corrections, only a few markers were significantly associated. We further applied the power of stepwise regression to identify the minimum number of markers for each rust pathogen race that explains nearly the same amount of variation as explained by all the markers considered together. Stepwise regression allows selection of markers from major QTL and makes it easy to choose a subset of markers to use in marker assisted selection [42].

Association analysis identified a total of 31 QTL markers in winter wheat landrace accessions associated with seedling resistance to leaf rust. These markers were located on chromosomes 1A, 1B, 2A, 3A, 3B, 4A, 4B, 5A, 5B, 6A, 6B, 6D and 7A. Of the 13 chromosomes that contained markers associated with resistance to one or more races of *Pt*, four chromosomes 3A, 4A, 5A, and 6D have not been previously shown to contain any leaf rust resistance genes originally from *T*. *aestivum* [44, Cereal Disease Lab, http://www.ars.usda.gov/main/docs.htm?docid=10342]. Therefore the eleven markers identified in genomic regions in 3A (47.75 cM, 72.50 cM, 118.07 cM), 4A (93.49 cM, 151.32 cM, 198.84 cM), 5A (53.71 cM, 87.89 cM, 122.72 cM) and 6D (45.42 cM, 69.20 cM) appear to be associated with novel sources of resistance and could be useful in breeding programs for seedling resistance to leaf rust (Table 4). Based on the general chromosome locations of previously identified leaf rust resistance genes in *T*. *aestivum* and their effectiveness on *Pt* races used in this study, markers identified in chromosomes 1A, 2A, and 4B could possibly be for *Lr10*, *Lr11* and *Lr30*, respectively. The markers identified in the other chromosomes could possibly be for seedling resistance genes *Lr31* (4BS), *Lr33* (1BL) and *Lr52* (5BS) that are not included in the differential set but have been previously identified in *T*. *aestivum* [47]. Comparison to a W7984/OpataM85 double haploid map integrating SSR, DArT, iSelect 9K SNP, and GBS markers [48] indicated that the markers IWA3160 and IWA435 associated with resistance to race TBDJ are located in the general chromosome region close to genes *Lr10* and *Lr33*, respectively. Similarly, the marker IWA6290 association for race TDBG is near the region where *Lr33* has been previously mapped [48]. To our knowledge, mapping information for *Lr11*, *Lr30*, *Lr31*, and *Lr52* is not available to allow for comparison with markers found in chromosomes where these resistance genes are located.

Three QTL were associated with resistance to *Pst* race PSTv-37. Two of these markers, IWA4240 and IWA7331 were located on the long arms of chromosomes 1A and 1B, respectively. Chromosome 1B contains several known stripe rust seedling resistance genes (*Yr3a*, *Yr3b*, *Yr3c*, *Yr10*, and *Yr21*) originating from *T*. *aestivum* [49–51]. *Yr10* is effective against PSTv-37, but is located on the short arm of chromosome 1B; therefore it cannot be responsible for the resistance response associated with the IWA7331 locus. Monosomic analysis by Chen et al. [51] showed that *Yr21* is located on chromosome 1BL. The *Yr3* alleles (*Yr3a*, *Yr3b*, and *Yr3c*) are not assigned to a specific chromosome arm [49]. Therefore, the association of marker IWA7331 with stripe rust resistance could possibly represent resistance genes *Yr3a*, *Yr3b*, *Yr3c* and *Yr21* or a new resistance locus. On chromosome 1A, only the temporarily designated seedling resistance gene *YrDa1* from *T*. *aestivum* has been previously identified, but not assigned to a specific chromosome arm [52] (Cereal Disease Lab 2014, http://www.ars.usda.gov/main/docs.htm?docid=10342). This suggests that IWA4240 could be a marker representing *YrDa1* or a novel resistance locus for *Pst* resistance. Further investigation using bi-parental population QTL mapping and additional comparative analysis as information becomes available, will provide more information about the relationship between *YrDa1* and the locus identified in chromosome 1AL.

The genome sequence of wheat is available but incomplete even with the rapid advancement in sequencing technology. This study searched for gene models in sorghum, rice, and *Brachypodium* that correspond to probe sequences for SNPs associated with leaf rust and stripe rust resistance (Table 4). Most of the sequences corresponded to putative proteins with enzyme activity such as protease (IWA2195), phosphatase (IWA7429, IWA574), and peptidase (IWA8186, IWA2126). However, several SNPs were found associated with genes that encode for putative proteins involved in disease resistance (R-proteins). IWA4240 on chromosome 1A, which is associated with PSTv-37 resistance, corresponded to a putative NB-ARC domain containing protein. The nucleotide binding (NB) domain is a domain found in the NB-LRR gene family which is often associated in plant disease resistance. The NB-ARC domain refers to the nucleotide binding domain of apoptotic protease activating factor 1 (APAF-1), R-proteins and *Caenorhabditis elegans* Death-4 (CED-4) [53]. Ooijen et al. [54] conducted a structured-function analysis and found that the NB-ARC is involved in regulation of R-protein. Another important R-protein domain, leucine-rich repeat (LRR), was associated with two SNP markers (IWA185 and IWA6340) and is part of the NBS-LRR superfamily that characterizes most R-proteins [55]. The LRR domain is mainly involved in recognition and interaction with other proteins in disease resistance pathways. Other important protein families associated with associated SNP markers include a protein kinase (IWA435), ABC transporters (IWA5005 and IWA5786), and zinc fingers (IWA8395, IWA3699 and IWA5000). Bruggeman et al. [56] found the Rpg1 protein for barley stem rust resistance to contain two tandem protein kinase domains. Similarly, the *Lr34* gene associated with durable resistance to leaf rust, stripe rust, and powdery mildew of wheat belongs to ABC transporter gene family [57]. Many proteins in the zinc finger superfamily are involved in biotic and abiotic stress in plants. Guo et al. [58] demonstrated that overexpression of a zinc finger protein, GhZFP1, enhanced tolerance to salt stress and resistance to *Rhizoctonia solani*.

The results of our study demonstrate the use of AM for the identification of potentially new genomic regions associated with leaf and stripe rust resistance that can help to broaden the genetic base of resistance in winter wheat breeding programs. The accessions identified in this study carrying resistance to multiple races and even both rust pathogens used in evaluations, could be excellent choices as parental lines in breeding programs interested in incorporating leaf and stripe rust resistance. Future work will focus on developing bi-parental populations of accessions to validate the resistance loci and develop user friendly, tightly linked markers that can be used to accelerate the incorporation of the novel resistance into elite breeding wheat lines.

## Supporting Information

S1 Table List of 567 winter-habit *Triticum aestivum* accessions along with their origin and linearized infection type to five *Puccinia triticina* (*Pt*) and one *Puccinia striiformis* f. sp. *tritici* (*Pst*) races. (XLSX)

S2 Table Properties for the 5,633 high quality SNP markers obtained from the wheat Illumina iSelect beadchip assay. (XLSX)

S3 Table List of resistant accessions, linearized infection type (IT), allele and chromosomal location for SNP markers associated with resistance. (XLSX)
